# The Effect of Anti-Autotaxin Aptamers on the Development of Proliferative Vitreoretinopathy

**DOI:** 10.3390/ijms242115926

**Published:** 2023-11-03

**Authors:** Hirotsugu Hanazaki, Harumasa Yokota, Satoru Yamagami, Yoshikazu Nakamura, Taiji Nagaoka

**Affiliations:** 1 Division of Ophthalmology, Department of Visual Sciences, Nihon University School of Medicine, 30-1 Oyaguchi-Kamicho, Itabashi-ku, Tokyo 173-8610, Japan; hanazaki.hirotsugu@nihon-u.ac.jp (H.H.); atokoy18@gmail.com (H.Y.); yamagami.satoru@nihon-u.ac.jp (S.Y.); 2 The Institute of Medical Science, The University of Tokyo, Minato-ku, Tokyo 108-8639, Japan; yoshi@ribomic.com; 3 RIBOMIC Inc., Minato-ku, Tokyo 108-0071, Japan

**Keywords:** proliferative vitreoretinopathy, retinal detachment, autotaxin, aptamer

## Abstract

This study investigated the effect of anti-autotaxin (ATX) aptamers on the development of proliferative vitreoretinopathy (PVR) in both in vivo and in vitro PVR swine models. For the in vitro study, primary retinal pigment epithelial (RPE) cells were obtained from porcine eyes and cultured for cell proliferation and migration assays. For the in vivo study, a swine PVR model was established by inducing retinal detachment and injecting cultured RPE cells (2.0 × 10^6^). Concurrently, 1 week after RPE cell injection, the anti-ATX aptamer, RBM-006 (10 mg/mL, 0.1 mL), was injected twice into the vitreous cavity. Post-injection effects of the anti-ATX aptamer on PVR development in the in vivo swine PVR model were investigated. For the in vitro evaluation, the cultured RPE cell proliferation and migration were significantly reduced at anti-ATX aptamer concentrations of 0.5–0.05 mg and at only 0.5 mg, respectively. Intravitreal administration of the anti-ATX aptamer also prevented tractional retinal detachment caused by PVR in the in vivo PVR model. We observed that the anti-ATX aptamer, RBM-006, inhibited PVR-related RPE cell proliferation and migration in vitro and inhibited the progression of PVR in the in vivo model, suggesting that the anti-ATX aptamer may be effective in preventing PVR.

## 1. Introduction

Proliferative vitreoretinopathy (PVR) is a severe form of retinopathy that causes irreversible blindness. The incidence of PVR progression after vitrectomy for rhegmatogenous retinal detachment is estimated to be approximately 10% [1,2,3]. In addition, the success rate of treatment for PVR is far lower than that for noncomplicated rhegmatogenous retinal detachment [2,4,5,6]. Therefore, novel strategies are required to prevent PVR following vitrectomy.

Fibrosis plays a crucial role in PVR development. Many studies have revealed that retinal pigment epithelial (RPE) cells play a significant role in fibrosis during the early stages of PVR [1,2,4,7,8]. RPE cells are dispersed through retinal breaks in the vitreous cavity and adhere to the retinal surface. Epithelial–mesenchymal transition (EMT) occurs in RPE cells, transforming them into fibroblast-like cells [9,10,11,12,13]. The interaction between fibrotic RPE cells and the extracellular matrix results in a proliferative membrane, which causes retinal traction and contraction and PVR. Although several studies have reported that various cytokines and growth factors are involved in PVR progression, the ideal target molecule to prevent this process remains undetermined.

Autotaxin (ATX), an enzyme that produces lysophosphatidic acid (LPA) from lysophosphatidylcholine (LPC), is widely distributed in the blood, cerebrospinal fluid, kidneys, lymphatic organs, and eyes [14,15,16]. LPA is a lysophospholipid that exhibits various effects, such as cell proliferation, migration, cell–cell adhesion, and fibrosis, by activating downstream pathways through interaction with its receptors. Recent studies have shown that the ATX–LPA pathway is activated in various glaucoma subtypes [17]. This pathway contributes to fibrotic changes and extracellular matrix production in the trabecular meshwork [18]. However, the role of the ATX–LPA pathway in PVR remains unclear.

Recently, anti-ATX aptamers have become available and have been shown to halt pulmonary fibrosis in mouse models of bleomycin-induced pulmonary fibrosis [19]. Aptamers are superior to antibody therapies in terms of target-binding power, lack of target restrictions, easy chemical modification, and low antigenicity [20,21,22].

In this study, we used an in vitro RPE cell culture from porcine eyeballs and an in vivo swine PVR model to focus on the ATX–LPA pathway in PVR development. We investigated whether anti-ATX aptamers have a beneficial inhibitory effect on this disease.

## 2. Results

### 2.1. RBM-006 Profile

RBM-006, the anti-ATX aptamer, bound stably and specifically to ATX; the binding of no other tested proteins, including heparin-binding proteins, was as stable. Importantly, RBM-006 blocked the LPC-to-LPA conversion activity of ATX with an IC_50_ of 0.5 nM. The half-life of RBM-006 in the blood was 3.7 h.

### 2.2. Anti-ATX Aptamer Inhibits RPE Proliferation

**Figure 1:**
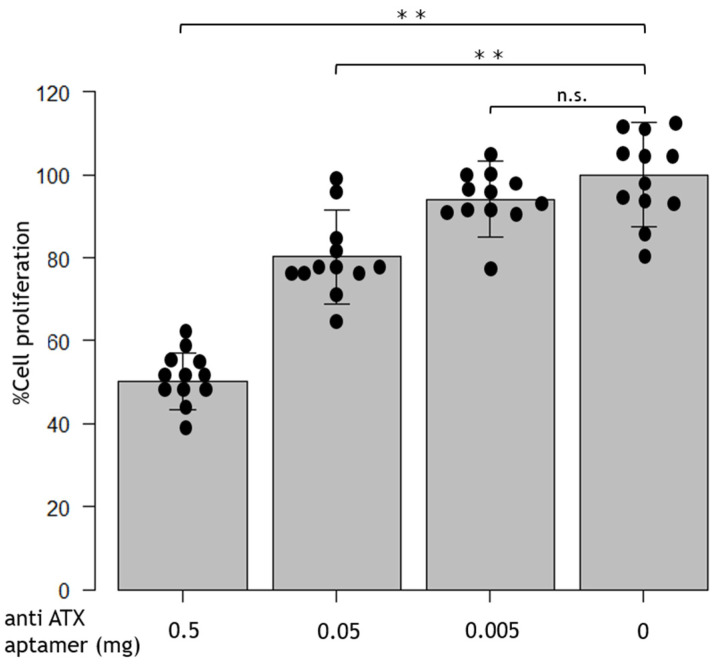
Effect of the anti-autotaxin (ATX) aptamer on retinal pigment epithelial (RPE) cell proliferation. There is no significant difference in the anti-ATX aptamer (RBM-006) at 0.005 mg, but cell proliferation significantly decreased at 0.05 and 0.5 mg compared with non-anti-ATX aptamer administration; the suppression occurred in a dose-dependent manner. ** *p* < 0.01; n.s., not significant. *n* = 12/group.

The anti-ATX aptamer RBM-006 was administered to cultured RPE (5.0 × 10^4^ cells), and cell proliferation was investigated. There was no significant difference at an anti-ATX aptamer concentration of 0.005 mg; however, cell proliferation significantly decreased when the aptamer was administered at concentrations of 0.05 and 0.5 mg compared with non-anti-ATX-aptamer-administered cells, with the suppression being in a dose-dependent manner (Figure 1).

### 2.3. Anti-ATX Aptamer Inhibits RPE Migration

**Figure 2:**
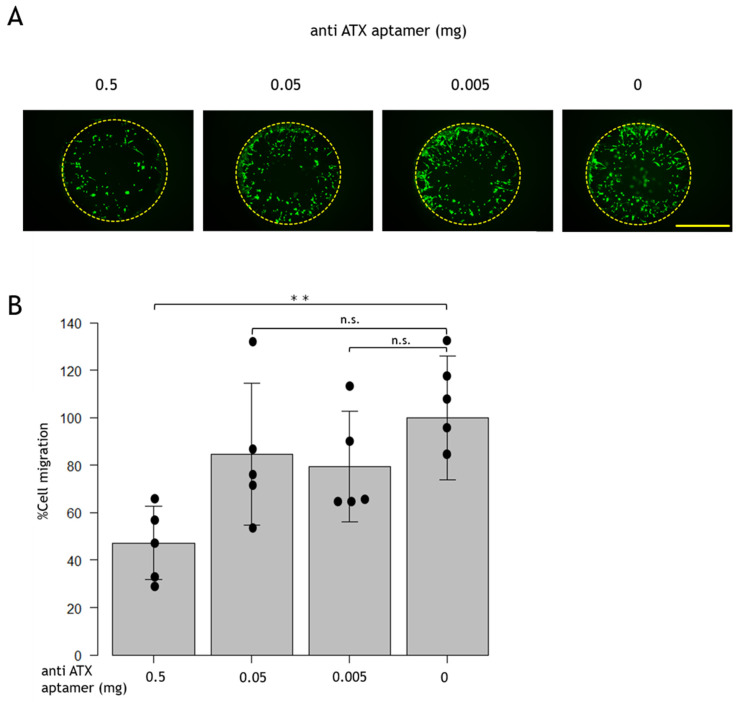
Effect of the anti-ATX aptamer on RPE cell migration. (**A**) Cell migration was measured in the defined zone (yellow dotted circle). Bar: 100 μm (**B**) No significant difference was observed between the anti-ATX aptamer (RBM-006) at 0.005 and 0.05 mg, but cell migration was significantly reduced at 0.5 mg when compared with non-anti-ATX-aptamer-administered cells. ** *p* < 0.01; n.s., not significant. *n* = 5/group.

We administered the anti-ATX aptamer (RBM-006) to cultured RPE cells (5.0 × 10^4^ cells) and examined cell migration. No significant difference was observed when the anti-ATX aptamer (RBM-006) was administered at concentrations of 0.005 and 0.05 mg, but cell migration was significantly reduced at 0.5 mg when compared with non-anti-ATX aptamer administration (Figure 2).

Normality was assessed with the Shapiro–Wilk test (cell proliferation, anti ATX aptamer (0.5 mg, 0.05 mg, 0.005 mg, 0 mg): *p* = 0.796, 0.095, 0.971, 0.363, cell migration; anti ATX aptamer (0.5 mg, 0.05 mg, 0.005 mg, 0 mg): *p* = 0.568, 0.369, 0.095, 0.982) and the equivariance of the distributions with the Bartlett test (cell proliferation: *p* = 0.235, cell migration: *p* = 0.665).

### 2.4. Anti-ATX Aptamer Inhibits the Progression of PVR In Vivo

**Figure 3:**
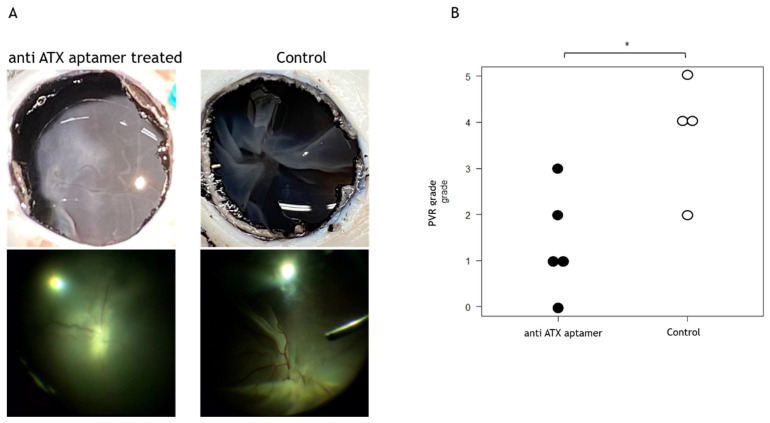
Effect of the anti-ATX aptamer on proliferative vitreoretinopathy (PVR) in vivo. (**A**) Administration of the anti-ATX aptamer suppressed the onset of tractional retinal detachment (TRD) in PVR. TRD occurred in PVR in controls without the anti-ATX aptamer. (**B**) The incidence of TRD in PVR was significantly reduced with anti-ATX aptamer administration compared with non-administration. * *p* < 0.05.

**Figure 4:**
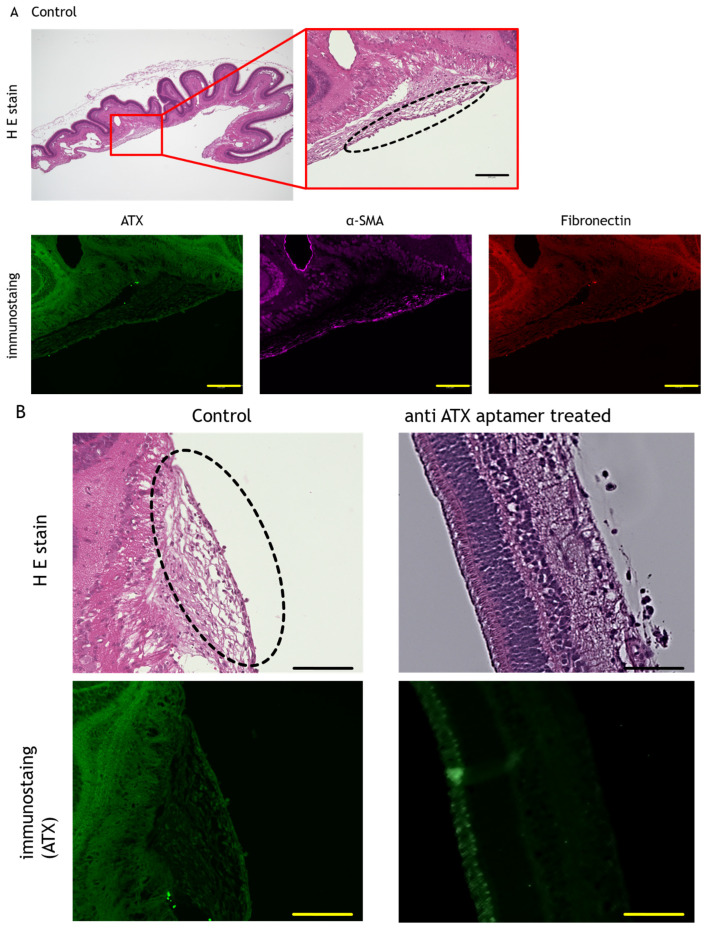
Histological image of anti-ATX aptamer administration. (**A**) Hematoxylin and eosin (HE) staining showing a proliferative membrane on the retina (black dotted circle) in the control. Immunostaining showing that the proliferative membrane is positive for ATX, α-smooth muscle actin (α-SMA), and fibronectin. (**B**) No proliferative membrane was seen after anti-ATX aptamer administration, and ATX positivity was not confirmed. Bar: 100 μm.

We administered 1.0 mg of anti-ATX aptamer (RBM-006) to cultured RPE (2.0 × 10^6^) cells on days 1 and 7. Cells without the administration of the anti-ATX aptamer were used as controls. The grade of PVR was confirmed on day 14. We found that the incidence of tractional retinal detachment (TRD) in PVR was significantly reduced in cells treated with RBM-006 compared with those without treatment (Figure 3). In the five eyes treated with the anti-ATX aptamer, no TRD of one or more quadrants was observed, except in one eye. In the control group, without the anti-ATX aptamer treatment, hematoxylin and eosin staining revealed a proliferative membrane on the retina. Immunostaining revealed that the proliferative membrane was positive for ATX, α-smooth muscle actin, and fibronectin. However, no proliferative membranes were observed after anti-ATX aptamer administration; ATX-positive tissues were not confirmed (Figure 4).

## 3. Discussion

In the present study, anti-ATX aptamer administration inhibited the proliferation and migration of RPE cells in vitro. Cell proliferation was significantly decreased at 0.05 and 0.5 mg anti-ATX aptamer administration compared with non-anti-ATX aptamer administration, and was suppressed in a dose-dependent manner (Figure 1). Compared with the non-anti-ATX aptamer administration, cell migration was significantly reduced at 0.5 mg (Figure 2). Thus, cell proliferation, migration, cell–cell adhesion, and fibrosis by EMT of the RPE, which are reported to be involved relatively early in the onset of PVR [9,10,11,12,13], have also been shown to play essential roles in the onset of PVR in our present study.

One of the RPE EMT mechanisms is transforming growth factor-β (TGF-β) signaling [9,23,24,25]. Glucosamine is known to inhibit the TGF-β signaling pathway in RPE cells and several downstream events associated with EMT [26]. Resveratrol also inhibits TGF-β signaling and suppresses the proliferation and migration of RPE [27]. TGF-β-induced EMT in RPE cells is greatly stimulated by fibroblast growth factor 2 in vitro and in vivo [25]. In addition, the Rho-kinase pathway is involved in cell proliferation, migration, cell–cell adhesion, and fibrosis. The inhibition of Rho-kinase decreases the contractile force generated by RPE cells and attenuates PVR [28,29]. LPA is expressed upstream of the TGF-β signaling and Rho-kinase pathways; it binds to extracellular receptors to regulate various physiological activities [30,31,32]. In previous studies, drug adjuvant treatments have been investigated regarding the downstream pathways of LPA. In contrast, we focused on ATX, an enzyme upstream of the LPA pathway, and investigated its effect. Anti-ATX aptamer administration inhibited the proliferation and migration of RPE cells.

LPA activation in mesothelial cells can stimulate cell proliferation and upregulate the expression of a profibrotic factor, connective tissue growth factor, in epithelial cells and fibroblasts [33,34,35,36,37]. The inhibition of ATX by the anti-ATX aptamer did not activate the downstream pathway of LPA and suppressed the proliferation and migration of RPE cells.

In the present study, administration of the anti-ATX aptamer also suppressed TRD in PVR (Figure 3). There are few previous reports on the use of swine models for PVR. Umazume et al. [38] reported that four intravitreal injections of dasatinib had inhibitory effects on PVR. We observed an inhibitory effect of PVR with intravitreal injections of two concentrations of the anti-ATX aptamer, which was less than that previously reported. When the drug is clinically applied as adjuvant therapy, it is advantageous for patients in whom a suppressive effect can be obtained with fewer administrations. This is because the more frequent the intravitreal injection, the higher the cost and the greater risk of complications such as endophthalmitis.

ATX expression has been observed on the proliferative surfaces in diseases such as proliferative diabetic retinopathy and PVR [39]. We also observed the presence of ATX on the proliferative membrane in a porcine PVR model in the absence of anti-ATX aptamer administration. In contrast, no proliferative membrane was observed with anti-ATX aptamer administration (Figure 4). These results suggest that ATX is involved in the pathogenesis of PVR and that ATX inhibition by ATX-aptamer suppresses the development of PVR.

Many previous studies have reported that EMT occurs in cultured RPE cells and that the EMT of RPE cells is an initial step in fibrotic processes such as cell proliferation, migration, and extracellular matrix remodeling in the pathogenesis of PVR [9,10,23,40,41]. The present findings indicate that anti-ATX aptamers may inhibit ATX and suppress cell proliferation, migration, cell–cell adhesion, and fibrosis downstream of the ATX–LPA pathway.

In this study, we used swine for the in vivo experiments. Swine eyes have many similarities to the human eye, such as size and anatomy [42,43]. Therefore, the effects of drugs in the swine model can be more easily applied to humans.

This study had several limitations. First, only a small number of swine were used because breeding swine is expensive and requires a lot of space. Recent ethical guidelines for experimental animals have made conducting research with a minimum number of samples necessary. Hence, the robustness of the data has been limited. Nevertheless, several previous studies with the swine model have proven useful to evaluate efficacy with a small number of samples [44,45,46], similarly to this study. Second, because the border between the proliferative membrane and retina is not necessarily clear, it was difficult to confirm the presence of a proliferative membrane on histological examination. Visualization of the proliferative membrane may be desirable, and future studies are warranted. Third, we could not examine the underlying mechanism and only confirmed the effectiveness of the anti-ATX aptamer in the present study. Further studies are needed to examine the downstream pathways of the ATX–LPA axis using inhibitors of TGF-beta or Rho-kinase and to assess the expression levels of relevant genes and proteins.

## 4. Materials and Methods

### 4.1. RBM-006 Profile

RBM-006 is an anti-ATX aptamer composed of 29 nucleotides, whose ribose 2′ positions are heavily modified to resist ribonucleases; the 5′- and 3′-termini are conjugated with 40 kDa polyethylene glycol and an inverted dT, respectively, to achieve sufficient pharmacokinetic profiles [47].

### 4.2. In Vitro RPE Cell Culture

We used 17 eyeballs from wild-type pigs for the in vitro experiments. RPE cells were isolated from porcine eyes (Tokyo Shibaura Organ Co. Ltd., Tokyo, Japan). Primary cultures of RPE cells were established following the previously described protocol [48]. The experiments were performed using RPE cells that had been grown at 2–6 weeks of age until they were confluent with a hexagonal shape and without visible pigmentation in 25 cm^2^ tissue culture flasks. The cells were cultured in Dulbecco’s modified Eagle medium containing glutamine (5 mL), penicillin/streptomycin (5 mL), and 5% fetal bovine serum.

### 4.3. In Vivo Swine PVR Model

We performed this study on nine eyes of nine wild-type pigs of both sexes (8–12 weeks old and 20–25 kg) (Zen-Noh, Tokyo, Japan). All animal procedures were approved by the Animal Care Committee of the Nihon University School of Medicine and were performed in accordance with the Association for Research in Vision and Ophthalmology Statement for the Use of Animals in Ophthalmic and Vision Research. Anesthesia was maintained through the inhalation of isoflurane (1–3%) during surgery. After sedation, pupils were dilated, and accommodation was relaxed with topical applications of 2.5% phenylephrine hydrochloride and 1% tropicamide.

**Figure 5:**
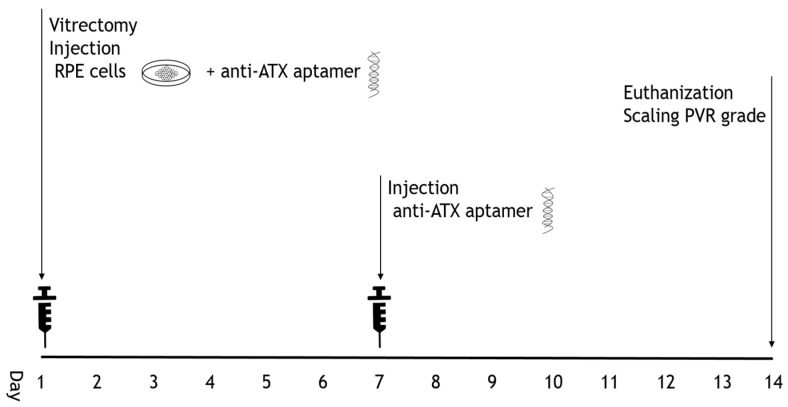
Protocol for the in vivo Swine PVR model. A vitrectomy was performed. At the end of vitrectomy, RPE cells were injected into the eye. Anti-ATX aptamer was also injected into the vitreous cavity after injecting RPE cells. Anti-ATX aptamer was re-injected 1 week after vitrectomy.

PVR was induced in swine using a three-step procedure, following the protocol reported by Umazume et al. [49]. The study protocol is summarized in Figure 5. Briefly, a three-port 25-gauge pars plana vitrectomy was performed, and the peripheral vitreous was shaved. Using a 39-gauge subretinal injection needle, a balanced salt solution was injected into the subretinal space to induce total retinal detachment. At the end of the vitrectomy, RPE cells (2.0 × 10^6^ cells) were injected into the eye. The anti-ATX aptamer RBM-006 (RIBOMIC, Tokyo, Japan) (10 mg/mL, 0.1 mL) (*n* = 5) or vehicle (*n* = 4) was injected into the vitreous cavity along with RPE cells. Anti-ATX aptamer RBM-006 or vehicle was reinjected 1 week after vitrectomy.

Clinical examinations were performed on postoperative days 7 and 14. PVR grading was based on a previously reported protocol [49].

### 4.4. Immunofluorescence and Immunohistochemistry

Swine were euthanized on day 14 with intravenous potassium chloride administered under general anesthesia and perfusion-fixed with 10% neutral-buffered formalin. After perfusion fixation, enucleation of the globe was performed, and the anterior segment was removed using a circumferential cut immediately posterior to the ora serrata. The eyecups were stored overnight in 10% neutral-buffered formalin. Paraffin sections were used for hematoxylin and eosin staining and immunostaining; section preparation was based on a previous protocol [25]. For immunostaining, primary antibodies for α-smooth muscle actin (#5694; Abcam, Cambridge, MA, USA) and fibronectin (#SC-8422; Santa Cruz Biotechnology, Dallas, TX, USA) for EMT markers and ATX (#D323-3; MBL, Tokyo, Japan) were used. Furthermore, Alex647-conjugated donkey anti-rabbit (Invitrogen), Alexa546-conjugated donkey anti-mouse (Invitrogen), and Alexa488-conjugated donkey anti-rat secondary antibodies (#150153; Abcam, Cambridge, MA, USA) were used for detection.

### 4.5. Cell Proliferation Assay

The proliferation of RPE cells was analyzed using a bromodeoxyuridine enzyme-linked immunosorbent assay (Roche Applied Science, Indianapolis, IN, USA), according to the manufacturer’s instructions, with or without the anti-ATX aptamer.

### 4.6. Cell Migration Assay

According to the manufacturer’s instructions, cell migration assays were performed using the Oris 96-well cell migration assay kit (Platypus Technologies, Madison, WI, USA). Cell migration was determined using Photoshop software (Adobe Systems, San Jose, CA, USA).

### 4.7. Statistical Analysis

The difference in PVR grading was analyzed using the Mann–Whitney U test, and one-way analysis of variance was used to analyze the in vitro assays (cell proliferation and cell migration). Normality was assessed with the Shapiro–Wilk test, and the equivariance of the distributions was assessed with the Bartlett test. All statistical analyses were performed using EZR software (Saitama Medical Center, Jichi Medical University, Saitama, Japan).

## 5. Conclusions

In conclusion, our findings showed that the anti-ATX aptamer inhibited RPE cell proliferation and migration in vitro in a dose–response manner. In addition, the intravitreal injection of RBM-006 (10 mg/mL, 0.1 mL) inhibited PVR progression in vivo. Our results suggest that anti-ATX aptamers may be effective in preventing PVR.

## Data Availability

The data presented in this study are available on request from the corresponding author.
